# Determining the Relationship between Mechanical Properties and Quantitative Magnetic Resonance Imaging of Joint Soft Tissues Using Patient-Specific Templates

**DOI:** 10.3390/bioengineering10091050

**Published:** 2023-09-07

**Authors:** Takehito Hananouchi, Shinji Satake, Kei Sakao, Hiroshi Katsuda, Nagakazu Shimada, Erik W. Dorthe, Darryl D. D’Lima

**Affiliations:** 1 Biodesign Division, Department of Academia-Government-Industry Collaboration, Hiroshima University, Hiroshima, Hiroshima 734-8551, Japan; 2 Department of Mechanical Engineering, Faculty of Engineering, Osaka Sangyo University, Daito, Osaka 574-8530, Japan; 3 Department of Orthopaedic Surgery, Shimada Hospital, 100-1 Kashiyama, Habikino, Osaka 583-0875, Japankeisakao626@yahoo.co.jp (K.S.);; 4 Shiley Center for Orthopaedic Research and Education at Scripps Clinic, La Jolla, CA 92037, USA

**Keywords:** quantitative MRI, mechanical property, joint soft tissue, articular cartilage, meniscus

## Abstract

To determine whether the mechanical properties of joint soft tissues such as cartilage can be calculated from quantitative magnetic resonance imaging (MRI) data, we investigated whether the mechanical properties of articular cartilage and meniscus scheduled to be resected during arthroplasty are correlated with the T2 relaxation time on quantitative MRI at the same location. Six patients who had undergone knee arthroplasty and seven who had undergone hip arthroplasty were examined. For the knee joint, the articular cartilage and lateral meniscus of the distal lateral condyle of the femur and proximal lateral tibia were examined, while for the hip joint, the articular cartilage above the femoral head was studied. We investigated the relationship between T2 relaxation time by quantitative MRI and stiffness using a hand-made compression tester at 235 locations. The patient-individualized template technique was used to align the two measurement sites. The results showed a negative correlation (from −0.30 to −0.35) in the less severely damaged articular cartilage and meniscus. This indicates that tissue mechanical properties can be calculated from T2 relaxation time, suggesting that quantitative MRI is useful in determining when to start loading after interventional surgery on cartilage tissue and in managing the health of elderly patients.

## 1. Introduction

Can the mechanical properties of cartilage and/or other soft tissues of the joints be calculated from quantitative magnetic resonance imaging (MRI) data? Quantitative MRI has been shown to indicate tissue quality. Specific tissue values have been calculated from the decay of intensity of several magnetic resonance images [1,2,3]. If the mechanical properties of the tissue, as one of the parameters of tissue quality, can be calculated from quantitative MRI data, confirming the mechanical properties of the surrounding tissue to be filled using preoperative quantitative MRI data in surgical intervention methods such as cartilage transplantation may be possible. Furthermore, noninvasively confirming the progress of the surgery and determining the optimal timing after such procedures, for example, when to start loading, may be possible.

At the moment, there are few studies on the relationship between quantitative MR imaging and the mechanical properties of articular cartilage and ligament in the knee joints. All the studies except one were conducted on human or animal cadaver tissues and the results identified some correlations [4,5,6,7].

When cadaver tissues (i.e., non-in vivo) are used for studies investigating the relationship between the mechanical properties and quantitative MRI of the target soft tissue, points on tissues resected from the cadaver are marked in advance, and the sites for investigation of quantitative MRI and mechanical properties are matched well [4,5,7]. To use the results of the above studies (i.e., There is a correlation between quantitative MR imaging and the mechanical property) in a clinical setting, the next step should be to investigate the correlation using in vivo data. However, in in vivo studies, marking the target tissue to analyze mechanical properties before MRI is not possible. One previous study conducted in a clinical setting found no correlation between quantitative MRI and mechanical properties; the study’s authors mentioned that the results could have been due to malalignment [6].

By creating a 3D model from preoperative images and 3D printing a mold that can conform to the surface of the model, a hole can be made in the planned direction during surgery [8,9,10,11] or a bone can be removed in line with a certain plane using the shape of the mold [12]. This technique is called “Individual template” [12]. This patient-specific template can be used to address the aforementioned issues. However, its use and usefulness in this type of experimental system have not been reported.

The purpose of this study was to investigate whether quantitative MRI data for joint soft tissue, which has been used in a clinical setting, correlates with mechanical properties investigated ex vivo as a preliminary step to accumulate reliable data for determining the relationship between quantitative MRI data and mechanical properties in clinical practice. For this purpose, we applied a patient-specific method to precisely align the points to be investigated in the quantitative MRI used in clinical practice with the points to be measured by the mechanical properties. We also investigated the relationship between them.

## 2. Methods

**Figure 1:**
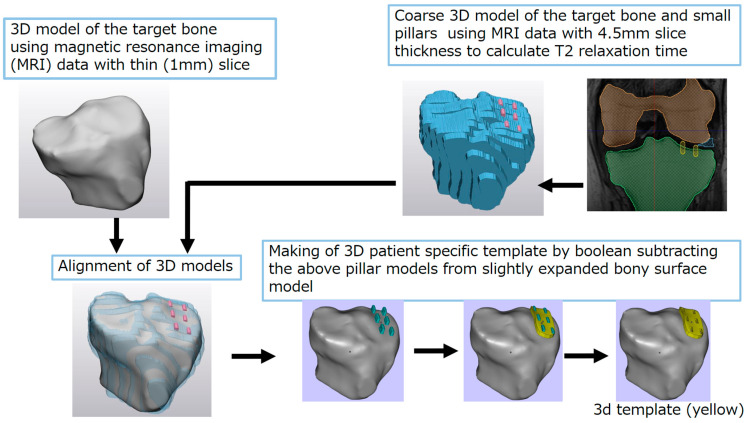
Process of creating an individual template using magnetic resonance imaging data.

Six female patients with knee osteoarthritis (OA) with a mean age of 73.6 years (range 58–84 years), mean height of 149.5 cm (range 144–153.3 cm), and mean weight of 59.3 kg (range 52.3–67.5 kg) who underwent total knee arthroplasty, and seven patients with hip OA (one male and six females) with a mean age of 67.5 years (range 60–73 years), mean height of 156.1 cm (range 150–84 cm), and mean weight of 60.3 kg (range 47–71.5 kg) who had undergone total hip arthroplasty were included in this study. The gender balance was not arbitrary. The surgeries were scheduled to allow the corresponding author to join the surgeries in the current studies. Of the patients in the surgeries, only one hip case was excluded due to the severe condition of the joint, which was classified as Grade 4 on the Kellgren and Lawrence system [13]. The study was approved by the Ethics Committees of Osaka Sangyo University (code: 2020-H.E.-02; date of approval: 2 June 2020) and Shimada Hospital (protocol code: 2020-011; date of approval: 20 May 2020), and the patients provided consent for participation in the study. The tissues used in the experiments were cartilage from the distally lateral femoral condyle and proximally lateral cartilage from the tibial surface and the lateral meniscus, which were removed during surgery, for the knee joint, and the cartilage on the surface of the femoral head for the hip joint. We investigated the correlation between the T2 value from the MRI scans and the mechanical properties of several locations of the tissues mentioned above. The process of conducting the compression study using the individual template technique is illustrated in Figure 1.

To create the template, a thin-slice MRI was required to reproduce the three-dimensional shape of the cross-section of the MR images. The knee joint was imaged along the coronal plane (repetition time [TR]: 30 ms; time to echo [TE]: 13.44 ms; slice thickness: 1.2 mm; slice interval: 0.6 mm; field of view [FOV]: 180) and the hip joint was imaged along the transverse plane (TR: 30 ms; TE: 12.62 ms; slice thickness: 1.4 mm; slice interval: 0.7 mm; FOV: 360). The images were imported into Seg3D (version 2.2.1), a segmentation software (URL; www.seg3d.org (accessed on 6 September 2023)) [14,15] to extract the bone contours, including cartilage, of the articular surfaces to create 3D models of the distal femur, proximal tibia, and lateral meniscus. Seg3D was developed by the Scientific Computing and Imaging Institute, University of Utah, USA, with funding from the National Institutes of Health.

For the quantitative MR images used to calculate the T2 relaxation time, we used coronal cross-sectional images with constant TR (knee: TR = 1500; hip: TR = 600) and eight TE times (knee: TE = 6.66, 13.33, 19.99, 26.66, 33.32, 39.98, 46.65, and 53.31 ms; hip: TE = 6.11, 12.22, 18.34, 24.45, 30.56, 36.67, 42.78, and 48.90 ms). The T2 relaxation time (T2 value) can be determined by the time constant of the return of the magnetic field from excitation to steady state. We used MRmap [16] to determine the T2 value. The knee joint was imaged with a slice thickness of 3 mm and a slice interval of 4.5 mm, and the hip joint was imaged with a slice thickness of 3 mm and a slice interval of 3.6 mm. The subsequent image processing was performed in Seg3D to record the coordinate information of the T2 value and to reflect it in the 3D model created.

To create a template to reflect the positional information of the T2 measurement points as small holes, we used 3-matic [17,18,19,20], a 3D CAD software (Materialise, Leuven, Belgium), to align the two aforementioned models of the same tissue but with different original data. In so doing, we also tracked the aforementioned column information (Figure 1). In the knee joint, this was performed for the femur, tibia, and meniscus, and in the hip joint, this was performed for the femoral head. The small columns could be displayed on the 3D model. Next, only the area where the small column exists partially protruded from the 3D model, and the template was created by performing a Boolean difference operation on the original shape (Figure 1) (Magics, Materilalise, Leuven, Belgium). This template (used as an individual template for each patient) was created using a 3D printer.

For total knee arthroplasty, a standard midline incision was performed in all patients using a medial parapatellar approach under an air tourniquet [21]. We then osteotomized the distal femur, which is perpendicular to the mechanical axis of the femur. The femoral bony surface was then extracted. After cutting the anterior and posterior chamfer and the intercondylar, the tibial bone was pulled anteriorly. The proximally tibial surface was then cut and extracted with meniscus. In total hip arthroplasty, a modified posterolateral approach was used [22], with dissection of the tensor fascia latae, split of the gluteus maximus, and incision of the short external rotators. After the hip capsule was opened, the femoral head was dislocated posteriorly, resected with a bone saw, and extracted from the surgical field. The extracted specimens were soaked in saline (Sodium Chloride, Otsuka Pharmaceutical Co., Ltd., Tokyo, Japan) and then stored in the refrigerator. The specimens were thawed before templating.

**Figure 2:**
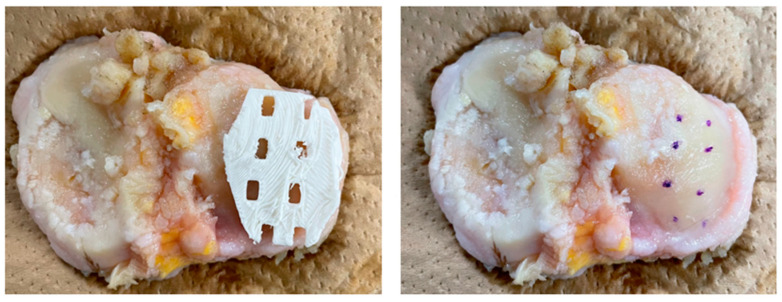
T2 relaxation time was reflected on the resected tissue with a template created using a 3D printer (**left**). A surgical pen was used to place markings through the holes in the template (**right**).

**Figure 3:**
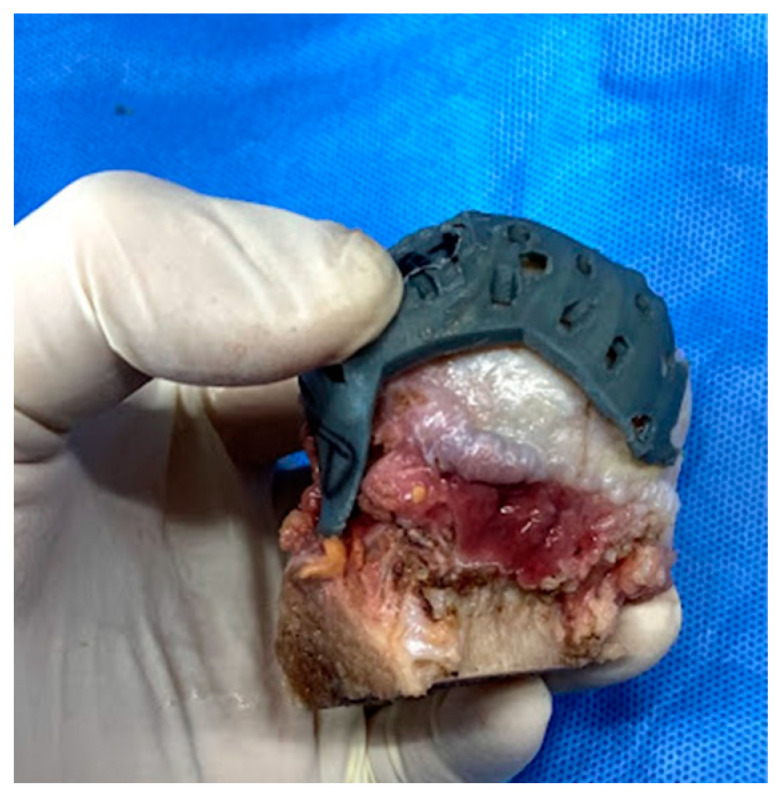
Individual template for the femoral head.

Each patient-specific template was placed on the specimen tissue to mark the surface of the tissue where T2 relaxation time was measured (Figure 2 and Figure 3). A surgical pen (Viscot Medical, LLC, East Hanover, NJ, USA) was used on the distal femoral and proximal tibial cartilage surfaces and on the lateral meniscus and femoral head cartilage surfaces to investigate the mechanical properties through compression testing, as described below.

**Figure 4:**
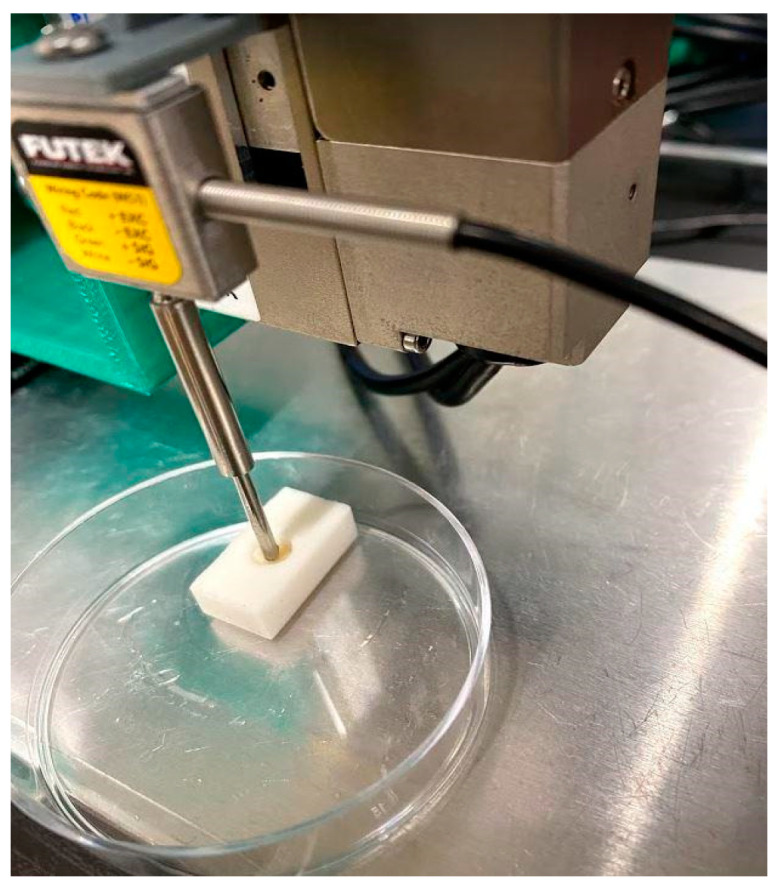
Investigation of the mechanical properties of the meniscus using a self-made compression testing apparatus.

Compression tests were performed in principle according to a previously described method using a self-made compression testing machine [7,23]. A 1-mm-diameter spherical indenter was used; the indentation displacement (distance) was 0.15 mm. As additional parameters in this analysis, the average difference between the two measurements of the compression test and the mimic cartilage samples [23] was 0.015 N/mm. The resolution of the load was 0.005 N. For the meniscus, a biopsy punch (φ = 5 mm, Kai industries Co., Ltd. Tokyo, Japan) was used to hollow out the marked area, and a hole of the same size was made on a plastic eraser (Radar, SEED Co., Ltd., Toyonaka, Osaka, Japan); the indentation displacement was 0.5 mm (Figure 4).

**Figure 5:**
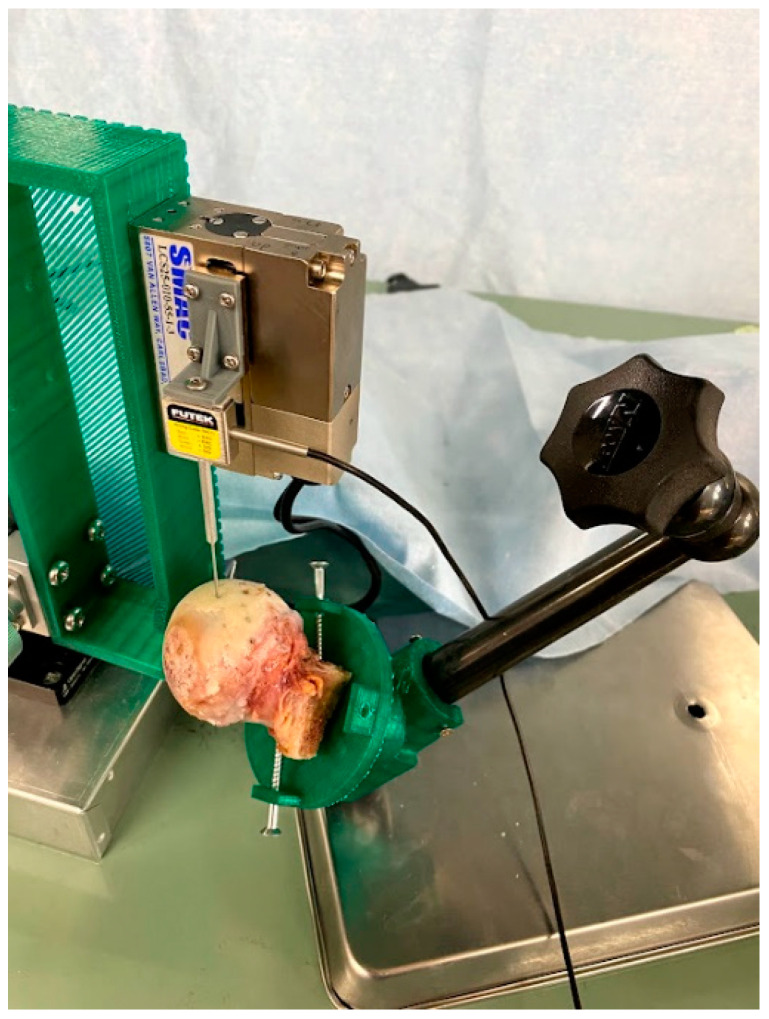
Compression test on cartilage tissue on the surface of the femoral head using a self-made device.

For the femoral head cartilage, a dial gauge stand (DG6160, Noga Waters, Ltd. Ueo, Saitama, Japan) and a base that could be attached to it were made using a 3D printer to adjust the surface to be measured to be perpendicular to the compression surface. The point to be measured was placed under the indenter. The displacement was 0.15 mm, similar to that for the knee joint (Figure 5). During the compression test, saline was applied to the entire surface using a syringe. Since the compression test was conducted for short periods at each interval, we believe that the effect of the evaporation of the water placed on the surface was negligible.

After calculating stiffness as a mechanical property using the compression test, the correlation (the Pearson correlation coefficient) between the stiffness and T2 value was calculated. For the distal femoral and proximal tibial articular cartilages of the knee joint and the femoral head cartilage of the hip joint, the degree of cartilage damage was classified by the corresponding author according to the International Cartilage Repair Society (ICRS) classification [24], and correlation was measured as a subgroup for each classification.

To verify the accuracy of the templates, we used five cases of resected tibial epiphyses after the completion of the compression test. The template was again placed against the articular surface, a homemade anchor made by a 3D printer company (TriMech Advanced Manufacturing Services (Formerly InterPRO), Deep River, CT, USA) was implanted through the holes of the template for fixation, and a computed tomography (CT) scan was taken. A postoperative bone model and a model of the template were created using Seg3D, and these models were superimposed on the preoperative model by 3-Matic to measure the absolute error in template position and the angular error of the template surface when observed along the coronal plane.

## 3. Results

The correlation between stiffness and T2 values was investigated. A total of 120 knee joint cartilage and 35 meniscus measurement points were identified. The correlation coefficients between stiffness and T2 values at each measurement point were −0.16 (*p* = 0.23) for the tibia, −0.20 (*p* = 0.13) for the femur, and −0.24 (*p* = 0.0095) for both the tibia and femur. Furthermore, the correlations for the grading of the cartilages in the knee joint were investigated by classifying them into four grades (grade I–IV); the results showed that grade II had higher correlations of −0.54 (*p* = 0.019) for the tibia, −0.39 (*p* = 0.0397) for the femur, and −0.46 (*p* = 0.001) for both the tibia and femur. In contrast, for grade III, the results showed very little correlation: −0.026 (*p* = 0.87) for the tibia, −0.054 (*p* = 0.76) for the femur, and −0.073 (*p* = 0.53) for both the tibia and femur (Table 1). The results also showed a statistically significant negative correlation for the meniscus (−0.36; *p* = 0.0328).

For the hip joint, analysis of the relationship between the T2 value of the cartilage surface of the femoral head and stiffness resulted in 83 measurement points. The overall correlation coefficient was −0.029 (*p* = 0.79).

**Figure 6:**
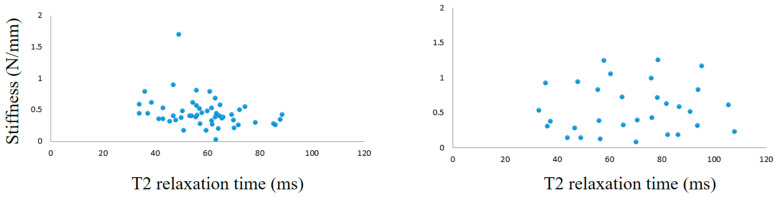
Correlations between T2 relaxation time and stiffness of the articular cartilage of the femoral head (**left**, grade II; **right**, grade III).

Grades II and III were identified based on the ICRS classification. Although the correlation coefficient for grade III (31 points) was unchanged at 0.07 (*p* = 0.70), the correlation for grade II (52 points) was negative, at −0.30 (*p* = 0.034) (Figure 6).

Regarding the accuracy of the templates, the position error was 0.2 ± 0.1 mm (range 0.1–0.3 mm) and the angle error was 0.9° ± 1.0° (range 0.01°–2.2°).

## 4. Discussion

Experiments were performed on intra-articular soft tissues (cartilage and meniscus) to investigate the relationship between the T2 value generated using the quantitative MRI technique and stiffness as one of the mechanical properties measured using the compression test. This template technique, which is used in many areas of orthopedic surgery, was used to align two locations to measure the mechanical properties of the measured location; its relevance was also investigated.

The results showed a negative correlation In the meniscus. In contrast, the articular cartilage showed no overall correlation for either the knee or hip joint; however, a negative correlation was found for grade II, that is, the part not severely damaged. This suggests that the T2 value can be used to calculate the strength of the meniscus and, to some extent, that of relatively undamaged cartilage. When the target tissue is damaged, water may enter the tissue, resulting in a change in the T2 values. Currently, quantitative MRI is used to evaluate damaged articular cartilage in many scenarios, including microfracture [25,26] and cartilage transplantation [27,28]. If strength can be predicted from T2 values, as indicated by the results of this study, determining the timing of load initiation may be possible.

Furthermore, if the intensity can be predicted from the T2 value, it can be used to guide some exercises in the elderly. Elderly individuals who are instructed to exercise to maintain their health may do so excessively, causing cartilage wear; thus, they should be instructed to exercise for appropriate amounts of time. If the images can predict intensity at this time, providing appropriate exercise guidance may be possible.

Although the relationship between cartilage strength and T2 values has been investigated, the relationship between these parameters and MRI values for in vivo knee cartilage tissues has not been properly evaluated [6]. The results showed no correlations; this may be because the alignment was inaccurate, as stated by the study investigators. To match the measurement points as closely as possible, the current study used the individual template technique, which uses the surface geometry of the target tissue.

The template technique has been used in various surgical procedures and has been reported to have higher accuracy, particularly in total knee arthroplasty and unicompartmental knee arthroplasty [29,30,31]. However, no studies have evaluated its accuracy using MRI data. The results of this study indicate that the placement was performed with high accuracy.

This study had some limitations. The sample size was small and only one type of quantitative MRI was confirmed. The results of indentation testing might also have some limitations. We did not evaluate the reproducibility of the indentation machine using the specimens in this study. However, we evaluated the reproducibility using the mimic cartilage tissue. In addition, indentation tests were performed ex vivo. Furthermore, the accuracy of the template was evaluated on the tibia only. Although the results were good, the impact of the spatial resolution of the MRI might affect the accuracy of the template technique. Further studies using larger sample sizes are required to determine whether the same results can be obtained with different types of quantitative MRI, such as T2 star [4].

## 5. Conclusions

To investigate the relationship between T2 value (relaxation time), a quantitative MRI, and stiffness, a mechanical property, we used a patient-specific template to align the above locations with good accuracy. The results showed negative correlations for less damaged cartilage tissue and the meniscus, suggesting that this imaging information is useful for determining the appropriate timing for the start of loading on cartilage tissue after interventional surgery or for making adjustments in directing the health of the elderly.

## Figures and Tables

**Table 1:** Pearson correlation coefficient between articular cartilage stiffness and T2 value in the excised knee joint tissue.

	Grade II	Grade III	Grade II & III
**Femur**	−0.39	−0.054	−0.2
**Tibia**	−0.54	−0.026	−0.16
**Femur & Tibia**	−0.46	−0.073	−0.24

## Data Availability

Not applicable.
